# Impact of Obesity on Anti-Mullerian Hormone (AMH) Levels in Women of Reproductive Age

**DOI:** 10.3390/jcm10143192

**Published:** 2021-07-20

**Authors:** Alexis L. Oldfield, Maryam Kazemi, Marla E. Lujan

**Affiliations:** 1 Biomedical and Biological Sciences, Cornell University, Ithaca, NY 14853, USA; alo49@cornell.edu; 2 Division of Nutritional Sciences, Cornell University, Ithaca, NY 14853, USA; maryam.kazemi@cornell.edu

**Keywords:** obesity, Anti-Müllerian hormone, ovary, body mass index, menstrual cycle

## Abstract

Obesity negatively impacts reproductive health, including ovarian function. Obesity has been posited to alter Anti-Müllerian hormone (AMH) production. Understanding biological factors that could impact AMH levels is necessary given the increasing use of AMH for predicting reproductive health outcomes in response to controlled ovarian stimulation, diagnosing ovulatory disorders, onset of menopause, and natural conception. In this narrative review, we evaluated the impact of obesity on AMH levels in healthy, regularly cycling reproductive-age women (18–48 years). Thirteen studies (*n* = 1214 women; (811, non-obese (body mass index; BMI < 30 kg/m^2^); 403, obese (BMI > 30 kg/m^2^))) were included, of which five reported decreased AMH levels with obesity, whereas eight showed comparable AMH levels between groups. Inclusion of women with higher obesity classes (Class 3 versus Class 1) may have been a factor in studies reporting lower AMH levels. Together, studies reporting AMH levels in otherwise healthy women remain limited by small sample sizes, cross-sectional designs, and lack of representation across the entire adiposity spectrum. Ultimately, the degree to which obesity may negatively impact AMH levels, and possibly ovarian reserve, in otherwise healthy women with regular menstrual cycles should be deemed uncertain at this time. This conclusion is prudent considering that the biological basis for an impact of obesity on AMH production is unknown.

## 1. Introduction

Obesity remains a persistent and growing public health concern, with current rates nearing 40% of reproductive-aged women in the United States [1]. Obesity impacts a broad array of health risks in women across the lifespan [2], including adverse reproductive health outcomes such as menstrual cycle irregularity, abnormal uterine bleeding, endometrial hyperplasia, infertility, and pregnancy complications [3,4,5,6,7]. Furthermore, women with obesity are 20% more likely to experience later onset of menopause, which in part may underlie the increased risk of breast, ovarian, and uterine cancer seen in this population [8]. While the impact of obesity on reproductive health is known to be multi-factorial, many of the adverse reproductive outcomes may be linked to endocrine disruptions that reflect an impaired ovarian function [9]. Specifically, infertility observed in women with obesity is commonly associated with ovulatory disturbances and irregular menstrual cyclicity [10]. However, even women with obesity and regular menstrual cycles exhibit a longer time to spontaneous pregnancy [11,12,13,14] and lower success rates of controlled ovarian hyperstimulation compared to their normal-weight counterparts [15]. This potential for subfertility aligns with previous reports of an altered reproductive hormone profile in women with obesity and regular cycles including, decreased follicle stimulating hormone (FSH) levels [16], decreased luteinizing hormone (LH) pulse amplitude [17], increased estradiol levels [16], and decreased luteal phase progesterone production [17]. Despite strides toward characterizing the nature of reproductive disturbances in obesity, several questions remain to be answered on how and why obesity may drive disordered ovarian function.

To that end, an altered ovarian follicular environment has been confirmed in women with obesity and involves disruptions in multiple systems, including steroidogenic action, metabolism, and inflammation, all of which can impact folliculogenesis and ovulatory potential [18]. The degree to which obesity impacts ovarian reserve is more controversial as available data have largely focused on sub- or infertile populations, wherein studies have not shown consistent associations between serum markers of ovarian reserve and body mass index (BMI) [4,19]. Anti-Mullerian hormone (AMH), a glycoprotein primarily produced by the granulosa cells of primary and early-stage antral follicles, is a marker whose association with obesity is controversial [20,21,22]—albeit a single meta-analysis suggests a negative association of AMH with BMI [19]. A growing interest in the use of AMH to predict reproductive health outcomes related to response to controlled ovarian stimulation [23], diagnosis of ovulatory disorders [24], the onset of menopause [25], and even natural conception [26] necessitate an understanding of biological factors, such as obesity, that could impact the predictive power of AMH for such reproductive outcomes.

The mechanisms through which obesity may adversely affect AMH production are unknown, but it has also been shown that with increasing adiposity, AMH production per antral follicle is reduced [27]. One possibility relates to an altered metabolic regulation of ovarian granulosa cells. Obesity is commonly associated with systemic insulin resistance and compensatory hyperinsulinemia. Excessive insulin levels have been shown to alter granulosa cell receptivity, and subsequently, AMH production [28]. Likewise, the increased leptin production associated with obesity could directly suppress AMH production. This observation is derived from the inhibitory effects of leptin administration on AMH and AMH receptor gene expression in cultured granulosa cells from patients undergoing controlled ovarian hyperstimulation [29]. More indirect in nature is the notion that lower AMH levels in women with obesity may result from a hemodilution effect of increasing body size [27]. Another possibility includes an impact of obesity on AMH catabolism and excretion. Obesity is known to alter the excretion of other reproductive hormones such as FSH, estradiol, and progesterone [17]. However, the exact mechanisms of AMH excretion are unknown [30]. Last, obesity may have an increased apoptotic effect at the ovarian follicle level, which is a mechanism observed in animal models [31]. While this posited mechanism may explain a reduced ovarian follicle pool and AMH levels, it seems less likely based on existing data of a later time to ovarian senescence in women with obesity.

Our current demographic necessitates further consideration of the impact of obesity on AMH production in healthy women of reproductive age. Most of the available data on AMH levels have been focused on women with infertility and/or polycystic ovary syndrome (PCOS) [32,33]. Of the data available in otherwise healthy women, AMH levels have been more commonly reported in women of lean BMI or women of advanced reproductive age [34,35]. Some women with obesity have regular cycles, yet their reproductive hormone profile suggests some level of ovarian dysfunction that could manifest as disordered AMH production compared to their lean counterparts [11]. Differences in AMH production across the adiposity spectrum could lead to inaccurate conclusions about the ability of AMH to adequately inform reproductive health outcomes in women. To address the current knowledge gap, we conducted a review to provide an up-to-date account of AMH levels in obese and non-obese women with regular menstrual cycles with the goal of establishing the degree to which obesity impacts AMH production in healthy, potentially fertile women.

## 2. Methods

This work represents a narrative review. The methods have been summarized herein.

### 2.1. Review Question

The PEO (Population (P), Exposure (E), Outcome (O)) criteria of our review were defined before the literature search. To that end, our study question was, in non-obese and obese regularly cycling women (P), are AMH levels (O) lower in reproductive age women with obesity and regular menstrual cycles compared to their non-obese counterparts (E)?

### 2.2. Primary Outcome

Our primary outcome was serum AMH levels.

### 2.3. Data Sources and Search Strategy

A search of published literature was conducted in the electronic databases of MEDLINE (PubMed), Institute for Scientific Information (ISI) Web of Science, and Scopus through 27 July 2020, using a search strategy based on the PEO framework, as described above. In short, studies included for review were limited to original research articles in which (1) the study was conducted in healthy, reproductive-aged (18–48 years) regularly cycling women, (2) the exposure was obesity, and (3) AMH levels were reported as an outcome for non-obese and obese groups. Only articles published in English were included. Studies must have used BMI as a categorical term, with obesity defined as a BMI > 30 kg/m^2^ and non-obese defined as some value < 30 kg/m^2^. Where AMH levels were reported separately for overweight women (BMI > 25 and <30 kg/m^2^), data were pooled with non-obese women where possible. Every record retrieved by this search strategy underwent a title and abstract screening to confirm that it aligned with the inclusion criteria. Articles that were relevant and appropriate were downloaded for full-text review, and data on the general characteristics of the study, patient population, study design, obesity definitions, AMH levels, and inclusion and exclusion criteria were extracted.

### 2.4. Inclusion and Exclusion Criteria

Briefly, observational (cross-sectional, case-control, cohort) studies or cross-sectional analysis of baseline measures from randomized controlled trials on women with regular menstrual cycles were included wherein the influence of obesity (non-obese and obese subtypes) as an exposure variable was evaluated on our study outcomes of interest. Non-peer-reviewed studies; studies without the design of interest; studies wherein our outcomes of interest were not compared between non-obese and obese women with regular cycles; studies that were not conducted on healthy women; studies in women with PCOS and women who had single isolated features of PCOS (hyperandrogenism, oligo- or amenorrhea, and polycystic ovarian morphology); studies featuring children (<17 years), pregnant women, or menopausal-aged women (>48 years); and, where study data were irretrievable after contacting their corresponding authors were excluded.

### 2.5. Data Extraction

The following data were extracted using a standardized protocol: (1) first author’s name; (2) study publication year; (3) participants’ characteristics, including total sample size and the sample size of participants in the non-obese and obese groups; (4) study design and setting and type of data analysis/collection (prospective/retrospective); (5) participants’ age; (6) participants’ body mass index (BMI); and (7) AMH levels.

## 3. Results

### 3.1. Literature Screening

One thousand nine hundred fifty-six studies were identified using the search strategy through electronic databases. Duplicates found using multiple databases, keywords, and sources were removed (*n* = 985). The titles and/or abstracts of the remaining records (*n* = 971) were screened, of which 615 studies were deemed irrelevant. The full texts of the remaining 356 studies were assessed for eligibility. Of these, 329 were further excluded due to the full text not being available (*n* = 7), study design not being appropriate (*n* = 105), no comparison between obese and non-obese women (*n* = 150), study not performed on reproductive-aged women (*n* = 51), AMH not reported as an outcome (*n* = 13), and duplicate reports of the same study data (*n* = 3). Twenty-seven studies remained, and 14 studies were further excluded due to an inconsistent definition of obesity. Ultimately, 13 studies were included in the review. A description of each study and its relevant characteristics and findings are summarized in Table 1.

### 3.2. Study Characteristics

Of the 13 studies identified in this review, eight involving a total of 193 obese and 261 non-obese women with regular menstrual cycles documented no significant differences in AMH levels between groups. Percent differences in AMH levels between groups ranged from −70.4% to 62.5% (Mean: −5.5%; Median: 2.5%). BMI of the non-obese participants ranged from 21.6 to 25.6 kg/m^2^, and BMI of the obese participants ranged from 31.7 to 34.3 kg/m^2^, which is consistent with the inclusion of women with strictly Class 1 (30 to <35 kg/m^2^) obesity. Studies were conducted across a broad array of countries and included diverse ethnic populations from North America [36,37], South America [38], Asia [39,40,41], and Africa [42,43]. Participants ranged in age from 23.8 to 46.2 years, with the mean age across studies being approximately 29 years. Studies were largely cross-sectional in nature and involved an assessment of serum AMH levels at a single time point during the menstrual cycle. The timing of the AMH assessment was not standardized to a particular stage of the cycle for all studies. However, six [38,40,42,44,45,46,47] of the 13 studies did measure AMH during the earliest part of the follicular phase (days 2–7). According to the most recent position statement by the American Society for Reproductive Medicine (ASRM), intracycle variation in AMH is considered minimal, and standardizing the timing of assessments is not a requirement at this time [48].

Five out of thirteen studies involving 210 obese and 550 non-obese women with regular menstrual cycles documented either significantly lower AMH levels in the obese compared to non-obese groups and/or a negative association between AMH and BMI. Percent differences in AMH levels between groups ranged from −9.7% to −76.7% (Mean: −27.4%; Median: −21.8%). The BMI of the non-obese participants ranged from 20.7 to 22.4 kg/m^2^, and that of the obese participants ranged from 33.0 to 46.0 kg/m^2^, which is consistent with the inclusion of women across Class 1 (30 to <35 kg/m^2^), Class 2 (35 to <40 kg/m^2^), and Class 3 (40 kg/m^2^ or higher) obesity—as well as a lack of any overweight individuals in the non-obese group. Studies were also conducted across a broad array of countries and included diverse ethnic populations from North America [46,47] and Europe [44,45,49]. Participants ranged in age from 23 to 46 years, with the mean age across studies being approximately 30 years. Studies were largely cross-sectional in nature and involved an assessment of serum AMH levels at a single time point during the menstrual cycle. Collectively, this group of studies included a similar number of obese women but more than double the number of non-obese women compared to the studies that reported no difference in AMH across BMI groups. A broader range of obesity was represented, but studies were more limited in their geographic representation.

**Table 1:** Characteristics of studies reporting AMH levels in non-obese and obese reproductive-aged women with regular menstrual cycles.

Lead Author, Publication Year (Country)	Participants’ Characteristics (n, Age (Year), BMI (kg/m^2^))	Group Definitions Based on BMI (kg/m^2^)	Study Design	Assay Type, Method	Cycle Day or Stage	AMH Levels			Correlation (*p*-Value) Adjustment for Confounders	Exclusion Criteria
						Obese	Non-Obese	*p*-Value across BMI Groups *		
Al-Eisa 2017 (Egypt) [43]	Non-obese group (*n*, 30; age, 28.7; BMI, 22.8) Obese group (*n*, 30; age, 27.6; BMI, 31.7)	Non-obese: 20–29 Obese: 30–35	Cross- sectional analysis of a non-randomized trial	Beckman Coulter ELISA	Day 2–3	4.60 (3.11–6.09)	2.83 (0.03–5.63)	>0.05	NR	Any PCOS feature, infertility, concomitant diseases, ovarian issues, use of drugs that affect hormone levels
Chiofalo 2017 (Italy) [49]	Non-obese group (*n*, 19; age, 30; BMI, 22) Obese group (*n*, 26; age, 33; BMI, 46)	Non-obese: <25 Obese: >30	Cohort	Gen II Beckman Coulter ELISA	Random	2.14 (0.81–3.47)	2.37 (0.17–4.57)	<0.0001	NR	PCOS, use of estroprogestin, metformin or inositol, hyperprolactinemia, and endocrine disorders
Eken 2019 (Turkey) [39]	Non-obese group (*n*, 38; age, 26.66; BMI, NR) Obese group (*n*, 31; age, 26.03; BMI, NR)	Non-obese: 18.5–24.9 Obese: >30	Cross- sectional	Ansh Labs AMH ELISA	Early follicular phase	2.56 (1.78–3.34)	2.30 (1.58–3.02)	>0.05	NR	PCOS, androgen-producing tumors, 21-hydroxylase deficiency, adrenal hyperplasia, hyperprolactinemia, thyroid disease, Cushing’s, smoking, and use of insulin sensitizers and/or medications that interfere with reproduction
Ersoy 2017 (Turkey) [41]	Non-obese group (*n*, 36; age, 26.4; BMI, 21.6) Obese group (*n*, 26; age, 26.7; BMI, 32.8)	Non-obese: 18.5–24.9 Obese: >30	Cross- sectional	Ansh Labs AMH ELISA	Day 2–4	3.10 (2.10–4.10)	3.10 (2.10–4.10)	NR	NR	PCOS, diabetes, Cushing’s, adrenal hyperplasia, androgen-secreting tumors, thyroid dysfunction, hyperandrogenism, hormonal drug use, and smoking, alcohol abuse
Halawaty 2010 (Egypt) [42]	Non-obese group (*n*, 50; age, 46.1; BMI, 25.6) Obese group (*n*, 50; age, 46.2; BMI, 32.9)	Non-obese: <30 Obese: 30–35	Prospective	DSL AMH ELISA	Day 2–5	2.55 (1.74–3.36)	3.39 (3.15–3.63)	0.56	NR	Use of hormones, smoking, pregnancy, lactation, hysterectomy, previous ovarian surgery, any PCOS feature, endometriosis, and other medical conditions that could affect ovarian function
Olszanecka-Glinianowicz, 2015 (Poland) [45]	Non-PCOS group (*n*, 36/67 obese; age, NR; BMI, NR)	Non-obese: 18.5–24.9 Obese: >30	Observational	Immunotech ELISA	Day 3–5	3.90 (1.60–6.20)	5.10 (2.70–7.50)	<0.05	−0.075 (*p* < 0.05) Age	Hyperandrogenism, PCOS, infertility, smoking, and alcohol use
Peigne 2020 (France) [44]	Non-obese group (*n*, 21; age, 32.0; BMI, 20.7) Obese group (*n*, 16; age, 31.5; BMI, 33.7)	Non-obese: <25 Obese: >30	Case-Control	DXI sandwich chemiluminescent immunoassay	Early follicular phase	0.87 API: 34.6%	0.92 API: 39.02%	*p* > 0.05 *p* < 0.001	NR API −0.557 (*p* < 0.01)	Any PCOS feature, use of medications that affect metabolism or ovarian function within 3 months
Roth 2014 (United States) [37]	Non-obese group (*n*, 10; age, 27.3; BMI, 22.3) Obese group (*n*, 10; age, 32.5; BMI, 34.3)	Non-obese: 18.5–25 Obese: >30	Cross- sectional	Gen II Beckman Coulter ELISA	Mid-cycle	0.02 (0.01–0.06)	0.05 (0.02–0.10)	0.10	NR	Hyperandrogenism, chronic diseases, use of exogenous sex steroids or medications known to affect reproductive hormones, regular exercise >4 h weekly, or attempting pregnancy
Shahin 2020 (Jordan) [40]	Non-obese group (NR) Obese group (NR)	Non-obese: 18.5–25 Obese: >30	Case-Control	Roche Cobas ECLIA	Day 2–4	3.11 (0.92–5.3)	2.91 (−0.16–5.98)	0.70	NR	PCOS, congenital adrenal hyperplasia, Cushing’s, malabsorptive or eating disorders, menopause, history of bariatric surgery
Shaw 2011 (United States) [36]	Non-obese group (*n*, 31; age, 23.8; BMI, 22.2) Obese group (*n*, 36; age, 27.3; BMI, 33.4)	Non-obese: <25 Obese: >30	Case-Control	Beckman Coulter ELISA	Random	0.64	0.61	0.76	NR	Post-menopause, breast cancer
Steiner 2017 (United States) [46]	Non-obese group (*n*, 461; age, NR; BMI, NR) Obese group (*n*,114; age, NR; BMI, NR)	Non-obese: 18.5–24.9 Obese: >30	Cohort	Gen II Beckman Coulter ELISA	Day 2–4	2.20 (0.90–4.00)	2.85 (1.50–5.50)	0.06	NR	Known fertility problems (sterilization, PCOS, tubal blockage), endometriosis, previous or current use of fertility treatments, partner with a history of infertility, lactation, recent use of injectable hormonal contraception
Su 2008 (United States) [47]	Non-obese group (*n*, 18; age, 45; BMI, 22.4) Obese group (*n*, 18; age, 45.1; BMI, 37.6)	Non-obese: <25 Obese: >30	Cross- sectional	DSL AMH ELISA	Day 1–4	0.07 (0.03–0.15)	0.30 (0.14–0.63)	0.01	*p* = 0.02	Hormonal therapy, contraception, PCOS
Woloszynek 2015 (Brazil) [38]	Non-obese group (*n*, 66; age, NR; BMI, NR) Obese group (*n*,10; age, NR; BMI, NR)	Non-obese: <25 Obese: >30	Cross- sectional	Gen II Beckman Coulter ELISA	Day 2–7	1.90 (0.40–10.90)	2.90 (0.30–11.20)	0.29	NR	Chronic diseases, menstrual irregularity, PCOS, infertility, hysterectomy, oophorectomy, serum LH and FSH concentrations out of the reference ranges

PCOS, polycystic ovary syndrome; BMI, body mass index; ELISA, enzyme-linked immunosorbent assay; NR, not reported; OCP, oral contraceptive pill; LH, luteinizing hormone; FSH, follicle-stimulating hormone. ECLIA; electrochemiluminescence immunoassay; API, AMH; prohormone index; AMH levels expressed as ng/mL. Mean (±SD) or Median (25–75th) are presented as provided by the manuscript. * Spearman’s correlation is presented where available.

## 4. Discussion

In the present work, we aimed to provide an updated review of whether obesity per se affects AMH production in healthy, potentially fertile women. Our study comprised a total of 13 studies involving 403 obese and 811 non-obese women, respectively. Overall, the data do not support a consistent impact of obesity on AMH levels in women with regular menstrual cycles. Specifically, of the 13 studies included in this review, only one was designed to evaluate the potential for differences in AMH levels between non-obese and obese women with regular menstrual cycles [42]. The remaining studies reported AMH levels for healthy non-obese and obese women with regular menstrual cycles as part of their (baseline) clinical characteristics for studies aimed at: (1) contrasting reproductive and metabolic features of women with or without PCOS [37,39,40,41,43,44,45,49], (2) predicting onset of menopause [17] or time to natural conception [26], or (3) assay validation and reference range development [38].

Our findings contrast those of Moslehi et al. [19], which is, to our knowledge, the only review exploring an impact of obesity on a broad array of ovarian reserve markers, including AMH. The authors showed lower AMH levels in obese women based on a pooled analysis involving 211 obese and 233 non-obese, fertile non-PCOS women (weighted mean differences, −0.94, 95% CI −1.14, −0.73 ng/mL) and a negative relationship between AMH and obesity [19]. Several reasons may have contributed to differences in the findings between Moslehi et al. and our current observations. Our approach to identifying relevant studies differed from that of Moslehi et al. in having used different definitions for non-obese and obese groups. We defined obesity as strictly being a BMI > 30 kg/m^2^ as per the World Health Organization (WHO) definition [50], whereas obesity was defined using variable cut-offs by Moslehi et. al. Furthermore, we included overweight women in the lean group when available. This was in contrast to Moslehi et al., who included overweight women in the obese group. Our decision to pool overweight women into the non-obese group was consistent with the approach taken by two of the included studies [42,43] and the observation that antral follicle dynamics and AMH production do not seem to be compromised during natural cycles in this population [51,52]. Most of the studies identified in our review (11/13 studies, 85%) [36,38,39,40,41,44,45,46,47,49,53] compared strictly obese (≥30 kg/m^2^) versus lean (<25 kg/m^2^) women, and only two studies [42,43] included overweight (25–29.9 kg/m^2^) women, which were grouped into their non-obese cohorts. Therefore, there are few data on AMH levels in overweight women included in this review reflecting that there are very few data available in this population at large. Unlike Moslehi et al., we did not include women with sub-fertility and accounted for more recent studies, since their search was limited to those published by 2016. Ultimately, we identified nearly twice as many studies (13 versus seven studies) that reported AMH levels in otherwise healthy reproductive-aged women with regular menstrual cycles, with a majority (54%) being published after 2016. Therefore, there was only an overlap of three studies between our reviews. Moslehi et al. noted significant heterogeneity in AMH levels as part of their evidence synthesis [19]. Yet unlike Moslehi et al., we decided not to conduct a pooled analysis owing to high heterogeneity in AMH levels in the studies we identified, as well as the high degree of heterogeneity in the approaches employed by the various studies. This is consistent with a recent approach used by others in their evidence synthesis of AMH levels in women with PCOS wherein experts concluded that heterogeneity across studies would not enable any robust conclusions to be drawn on pooled analyses involving AMH [54].

### 4.1. Most Studies Showed No Difference in AMH Levels with Obesity

Included in this group of eight studies was the lone study whose primary aim was to evaluate differences in AMH levels between obese (*n* = 50) and non-obese (*n* = 50) groups. Mean AMH levels were 32.9% lower in the obese group compared to the non-obese group, but differences did not reach statistical significance [42]. While this study used stringent criteria to corroborate the healthy reproductive status of the participants, Halawaty et al. used a narrow definition for obesity (30–35 kg/m^2^), which primarily included women with Class 1 obesity. Furthermore, the mean and range of the BMI of the non-obese group were 25.6 and 24–29 kg/m^2^, respectively, possibly indicating a small number of women with BMI 18.5–24.9 kg/m^2^ in the lean group. Ultimately, the spectrum of adiposity in the study by Halawaty et al. may not have been sufficient to capture a significant effect of obesity on AMH production [42]. It must also be noted that this study focused on establishing an impact of obesity on the markers of ovarian reserve, specifically in older reproductive-aged women during the early transition phase of the late premenopausal state. As such, all women demonstrated regular menstrual cycle length (22–35 days) but also variability in cycle length by seven days in either direction for at least two cycles. The mean age of the non-obese and obese groups was 46.1 and 46.2 years and may not wholly reflect AMH production in younger women that are well outside the perimenopausal transition.

In a study by Woloszynek et al. involving 100 younger women with a mean age of 31, AMH levels were not significantly different across three BMI categories (lean, overweight, and obese) [38]. The study was designed to validate the use of the Gen II AMH immunoassay for use in reproductive-aged males and females and develop normative reference ranges for these populations. As secondary aims, any potential influence of hormonal contraceptives, smoking, and BMI on AMH levels was evaluated. Woloszynek et al. did not detect a significant association between AMH and BMI even after accounting for age in their analysis. This study involved a small number of women with a BMI > 30 kg/m^2^ (n = 10), and the AMH values for this group were highly variable (95% CI: 0.4–10.9 ng/mL), which is in line with its minimal contribution to the pooled analysis. Furthermore, approximately 30% of all females included in the study used oral hormonal contraceptives (OCP), albeit the degree to which the groups of interests were on OCP was not reported. The unbalanced representation of obesity in this cohort alongside any confounding effects of OCP use may have impacted the ability to detect an impact of obesity on AMH levels.

### 4.2. Fewer Studies Showed Decreased AMH with Obesity

Except for a single study [48], the remaining four studies included in this group were small, involving ≤50 participants in both non-obese and obese cohorts combined. While women in the obese and non-obese groups across all these studies had comparable age distributions, the BMI classes of the groups were variable, especially in those with obesity, and none of the studies included women who were overweight. Of these, the studies by Chiofalo et al. [49] and Olszanecka-Glinianowicz et al. [45] showed significantly lower AMH levels in obese versus non-obese women, with AMH levels being 9.7% (*p* < 0.0001) and 23.5% (*p* < 0.01) lower, respectively. Furthermore, the study by Olszanecka-Glinianowicz et al. showed a negative correlation between AMH levels and BMI (r = −0.30, *p* < 0001). Chiofalo et al. evaluated AMH levels as part of an intervention study involving bariatric surgery. As such, their obese group consisted of women with Class 3 obesity (mean BMI = 46 kg/m^2^). In contrast, the study by Olszanecka-Glinianowicz et al. that investigated AMH levels in the context of largely Class 1 obesity. Overall, these results suggest that obesity may have a negative impact on AMH across the obesity spectrum with a dose effect that is not linear.

Furthermore, a small study (*n* = 36), Su et al. (2008) examined associations between obesity and serum and ultrasound measures of ovarian reserve in women of late reproductive age (mean age: 45 years) who did not use hormonal contraceptives or have PCOS [47]. AMH levels were a striking 76.7% lower in the obese cohort compared to the non-obese group (*p* = 0.014). The authors identified BMI as an independent predictor of AMH and concluded that lower AMH levels in obese women of late reproductive age resulted from physiologic processes other than a decreased ovarian reserve.

Of the studies with larger sample sizes, Steiner et al. reported a trend (*p* = 0.06) toward differences in AMH levels across BMI groups involving a total of 750 women in underweight, lean, overweight, and obese groups [46]. In the case of groups of interest to this review, AMH levels were 29.5% and 28.1% lower in 114 obese women with regular cycles and no history of infertility compared to 461 lean and 155 overweight women with similar reproductive health histories, respectively—which is consistent with AMH levels being quite similar in lean and overweight groups. The study was designed to assess any association between the biomarkers of ovarian reserve and time to natural conception in a group of late reproductive age women (30–44 years) in which rigorous approaches were used to exclude known fertility problems, ovaries disorders, and recent hormonal conception use. Ultimately, Steiner et al. adjusted their time to pregnancy models for AMH by BMI to reflect obesity as an important covariate.

### 4.3. AMH Assay Variability as the Main Factor Contributing to Inconsistent Reports

Differences in AMH levels between healthy women and women with subfertility have been well documented [22]. This difference is reflected in other markers of ovarian reserve, such as antral follicle counts [55]. Biological factors, including age, genetic variation within the AMH gene (i.e., *AMH* and *AMH2*) [56], race and ethnicity [57], and stage of the menstrual cycle [58] all have been posited to influence AMH production to varying degrees, with some factors garnering more support than others. By contrast, technical factors related to variability in AMH assay performance are an accepted contributor. In our review, seven different assays were used across the 13 included studies. Comparability studies have been performed between some of the assays used but given the large number of AMH assays on the market and the discontinuation of others, not all the assays represented in this review have been evaluated against each other for comparability. Namely, data are available to contrast the performance of the original Beckman Coulter ELISA, Beckman Coulter Gen II ELISA, Roche Elecys Cobas, and the Ansh ELISA. However, data are not available for the DSL ELISA, Immunotech ELISA, or DXI sandwich chemiluminescent immunoassay. Based on the available evidence, AMH levels measured by the different assays can vary drastically (up to 40%) [59]. This appreciable sample-to-sample variability and the substantial discrepancies in the between-assay conversion factors that have been proposed suggest assay performance issues. Differential responses to pre-analytical proteolysis, conformational changes of the AMH dimer, or the presence of interfering substances are speculated to play a role [54]. To that end, Teede et al. recently called for an international reference standard for AMH and the more robust independent evaluation of commercial assays in routine use using clinical samples with well-defined sample handling and processing protocols [54].

### 4.4. Other Factors Contributing to Inconsistent Reports

This review centered on women with regular menstrual cycles. Yet, in line with some of the studies included in this review, we cannot exclude the possibility that some women may have had a mild variant of PCOS according to the Rotterdam criteria (i.e., hyperandrogenism and polycystic ovarian morphology). Eleven of the 13 studies identified in this review explicitly used a PCOS diagnosis as an exclusionary factor, and we were careful to exclude studies wherein subfertility or infertility were documented in the control group, which together make the inclusion of women with mild PCOS unlikely. That being said, inclusion of women with milder forms of PCOS could confound the interpretation of AMH levels given that AMH is known to be elevated in PCOS [24]—albeit a negative relationship between AMH and BMI has been observed in the context of PCOS [60]. Another important confounder is the use of hormonal contraceptives. Nine of the 13 studies excluded women using medications known to interfere with reproduction, such as oral contraceptive pills (OCP) or other forms of hormonal contraception. However, four studies made no mention of their use. Furthermore, of the nine studies that excluded for OCP use, they did so only in the context of recent use (i.e., in the last 2 months). Studies have shown that women on OCP or other hormonal contraceptives display lower AMH levels [61,62] with long-term users having 30% lower AMH levels than non-users [63,64]. This is an important consideration in the context of this review, since hormonal contraception is known to have variable responses in obese populations [65]. Lastly, it is well documented that a history of smoking is associated with decreased AMH levels, with one study reporting up to a 4-fold decrease [61]. Only four of the papers included in the review specifically excluded women who were smokers. As such, any effect of smoking may be present in this analysis, confounding the impact of BMI on AMH. Future research should account for these important variables.

## 5. Conclusions

Collectively, the findings of this review do not corroborate a consistent negative impact of obesity on AMH. Our evaluation is consistent with most studies showing comparable AMH levels between obese and non-obese groups of reproductive-aged women with regular menstrual cycles, wherein women with obesity primarily presented with milder obesity status. Studies reporting AMH levels in otherwise healthy women are primarily limited by small sample size, cross-sectional design, and lack of representation across the entire adiposity spectrum—with an existing emphasis on lower obesity classes and absence of women who are overweight. Given that previous evidence supports that severe obesity may lower AMH levels and possibly ovarian reserve, it is critical to address this knowledge gap [27]. Few studies have prospectively evaluated the relationship between obesity and AMH production, and more mechanistic studies are needed to better understand how obesity and/or alterations in metabolic status could regulate AMH. The inclusion of women across all BMI classes (including overweight and Class 1–3 obesity) should be prioritized in future research with larger samples sizes to ensure the generalizability of findings to all reproductive-aged women. Ultimately, the notion of a negative impact of obesity on AMH in healthy women with regular menstrual cycles may be deemed uncertain at this time—or at best limited to women with severe obesity, warranting further investigations to address the limitations of the current evidence. This conclusion is prudent in light of the biological basis for such an impact being largely unknown.

## Data Availability

No new data were created or analyzed in this study. Data sharing is not applicable to this article.
